# A New Feature Extraction and Recognition Method for Microexpression Based on Local Non-negative Matrix Factorization

**DOI:** 10.3389/fnbot.2020.579338

**Published:** 2020-11-16

**Authors:** Junli Gao, Huajun Chen, Xiaohua Zhang, Jing Guo, Wenyu Liang

**Affiliations:** ^1^School of Automation, Guangdong University of Technology, Guangzhou, China; ^2^College of Automation, Zhongkai University of Agriculture and Engineering, Guangzhou, China; ^3^Institute for Infocomm Research (I2R), Agency for Science, Technology and Research (A*STAR), Singapore, Singapore

**Keywords:** macro-expression, micro-expression, macro-to-micro transformation, feature extraction, non-negative matrix factorization, CK+/CASME2/SAMM datasets

## Abstract

Microexpression is usually characterized by short duration and small action range, and the existing general expression recognition algorithms do not work well for microexpression. As a feature extraction method, non-negative matrix factorization can decompose the original data into different components, which has been successfully applied to facial recognition. In this paper, local non-negative matrix factorization is explored to decompose microexpression into some facial muscle actions, and extract features for recognition based on apex frame. However, the existing microexpression datasets fall short of samples to train a classifier with good generalization. The macro-to-micro algorithm based on singular value decomposition can augment the number of microexpressions, but it cannot meet non-negative properties of feature vectors. To address these problems, we propose an improved macro-to-micro algorithm to augment microexpression samples by manipulating the macroexpression data based on local non-negative matrix factorization. Finally, several experiments are conducted to verify the effectiveness of the proposed scheme, which results show that it has a higher recognition accuracy for microexpression compared with the related algorithms based on CK+/CASME2/SAMM datasets.

## 1. Introduction

Expression is one of the important ways for human to communicate emotion. In 1970s, American psychologist Paul Ekman defined six basic expressions of human, namely, happiness, anger, surprise, fear, disgust, and sadness. Facial expression recognition is to extract the specific states from given images or video, then identify the psychological emotions of the recognized object and understand its facial expressions. Expression recognition has many applications in psychology, intelligent monitoring, robotics, etc. Moreover, sometimes people may disguise their emotion and expression for various purposes. However, people cannot completely suppress their emotions under external strong emotional stimulus. There are some subtle and fast facial actions, which were first discovered and named “micro-momentary” expressions by Haggard and Isaacs (1966). Then, Ekman and Friesen formally named them microexpressions (Ekman and Friesen, 1969). It is an uncontrolled expression, which can be classified into the six basic emotions (Wu and Fu, 2010).

Different from the general expression, the microexpression is only reflected in a few facial action units, and the duration is about $\frac{1}{12}$ to $\frac{1}{2}$ s, which is difficult to detect. In addition, microexpression usually appears when people try to cover up their emotions. It is one kind of subconscious, inside-to-outside, uncontrolled, and undetectable behaviors with naked eyes. Because microexpression cannot be hidden, people can exploit this kind of weak, partial, short-term behaviors to acquire the hidden real emotions. Notably, microexpression recognition has many valuable applications in psychology, clinical diagnosis, business negotiation, interrogation, human–robot interaction, and so on, but it needs special training to master relevant recognition skills. In 2002, Ekman developed microexpression training tool (METT) (Ekman, 2006) that can train the recognition skills of the six basic emotions and other kinds of expressions, such as contempt, pain, and so on. At first, the recognition abilities of observers are tested through METT, then related knowledge of microexpression recognition is taught. After repeated training and consolidation, the recognition accuracy of observers can be improved by 40%. Nevertheless, the accuracy may be affected by various subjective factors, such as mood or preconceived thinking of the observers.

Microexpression is weak, short term, and difficult to detect, so the traditional expression recognition algorithms do not work well at all for this task. Generally, microexpression recognition can be divided into detection and classification. The former is to determine whether there are microexpressions in an image sequences, and detect the start/apex/end frames of a microexpression. The latter includes feature extraction and classification, which is similar to the general tasks of pattern classification. Significantly, the feature extraction is to acquire the abstract information from the data, which usually is some vectors obtained by image processing. The related algorithms can be used for extracting features, which can reflect the microexpression action information and distinguish various kinds of emotions. The feature classification is to train a classifier on the obtained vectors, directly related to the recognition accuracy, to distinguish the types of microexpression.

The main contributions of this paper are summarized as follows: (i) A local non-negative matrix factorization (LNMF) is developed to extract the features of apex frame on microexpression, which exploits local properties of LNMF to reflect the features of local action on microexpression. (ii) An improved macro-to-micro (MtM) transformation algorithm is proposed to augment the samples of microexpressions from macroexpression data based on LNMF. (iii) The performance of the proposed scheme is verified on CK+, CASME2, and SAMM datasets, which can benefit this work on human–robot interaction.

The rest of the paper is organized as follows. Related works are discussed in section 2. In section 3, the overall scheme, including theoretical derivation on LNMF and MtM algorithm design, is presented. Section 4 provides the experimental process and result analysis. Finally, we conclude this paper in section 5.

## 2. Related Work

Local binary pattern (LBP) is a commonly used method for extracting texture feature of images. LBP from three orthogonal planes (LBP-TOP) is an extension of LBP in video data. Ojala et al. (2002) and Zhao and Pietikainen (2007) acquired the feature vectors of the whole video by extracting the XY, XT, YT plane features of video. Yan et al. (2014) used LBP-TOP to extract the features of cropped face video in CASME2, and take support vector machine (SVM) as the classifier to recognize five categories of expressions with an accuracy of 63.41%. To reduce information redundancy and computational complexity of LBP-TOP, Wang et al. proposed LBP six intersection points (LBP-SIP) feature extraction algorithm (Wang Y. et al., 2014). Ben et al. (2018) proposed the second-order descriptor hot wheel patterns from TOP (HWP-TOP). It adopts 16 points on the inner and outer circles for calculation to extract more abundant feature information instead of eight points around the center pixel used by LBP.

Optical flow method aims to quantify facial muscle actions by calculating the motion speed of each pixel in the video. On this basis, the optical strain that reflects the distortion caused by small area motion can be further calculated. If the speed of a pixel in the image is higher than that of the surrounding pixels, its optical strain value will be higher, which can be used to detect the fast and micromovement of muscles in microexpression recognition. Liong et al. (2014) used the optical strain feature weights to highlight the features of the moving area. Liu et al. (2016) proposed the main directional mean optical flow (MDMO), which takes into account the local facial spatial position, statistical motion features, and has lower feature dimension to improve the calculation efficiency. Liu et al. (2018) also proposed the sparse MDMO to solve the problems that average operation in MDMO may lose manifold structures in feature space. Moreover, Wang et al. (2014a) took microexpression video as a fourth-order tensor, and proposed the tensor-independent color space (TICS) algorithm. They also extracted microexpression, and exclude irrelevant images to conduct the recognition through low-rank decomposition of samples (Wang et al., 2014b).

To determine the facial range of feature extraction, Liong et al. (2018b) counted the action units corresponding to all kinds of microexpressions. They found that the actions are only concentrated in a few facial areas, especially in eyes and mouth. If only the features of these three regions are extracted, irrelevant facial information can be filtered out and detection accuracy can be improved effectively. Therefore, this paper determines the region of interests (RoIs) of feature extraction through the distance between inner eyes and mouth corners. As the objects of feature extraction, most of the work directly calculate features of the whole video segment (Chen et al., 2019; Cao et al., 2021), while the apex frame contains the main information of microexpression (Li et al., 2018; Liong et al., 2018a). The apex frame refers to the moment when the movement amplitudes of the facial action units reach peak value in the duration of microexpression. Obviously, only extracting the features of apex frame can dramatically decrease calculating and eliminate the interference caused by irrelevant information in the video, which is also the basis of this paper.

Matrix factorization is popular in dimension-reduction fields, which has good physical significance. The original data are expressed as the weighted sum of several bases, which is transformed into a feature vector including weight coefficients to realize perception of the whole from local parts. Principal component analysis (PCA) and singular value decomposition (SVD) are the classic matrix factorization methods. However, the bases and coefficients calculated by these algorithms contain negative elements, which make the decomposition results not well-interpreted. For example, it is not practical to decompose face images into basic sub-images with negative components. To solve this problem, Lee and Seung (2000) proposed the non-negative matrix factorization (NMF) based on non-negative constraints of matrix elements. Li et al. (2001) pointed out that the bases calculated by NMF are redundant and not independent. Hence, the local constraints were added during calculation, that is, LNMF was proposed. The local action of microexpression can be reflected by the local features of LNMF, which is also the reason why we adopt LNMF to extract features of microexpression.

Nowadays, CASME (Yan et al., 2013), CASME2 (Yan et al., 2014), CAS(ME)2 (Qu et al., 2016), and SAMM (Davison et al., 2018a,b) are the widely used datasets for microexpression recognition and classification. However, each dataset has only hundreds of samples, and the number of different microexpressions is seriously unbalanced, which is not sufficient to train a classifier with better generalization ability, especially for deep neural network (DNN). Naturally, researchers hope to train microexpression classifiers by means of numerous macroexpression datasets (Wang et al., 2018; Peng et al., 2019; Zhi et al., 2019). Jia et al. (2018) proposed an MtM algorithm, which uses macroexpression data to generate microexpression samples by constructing corresponding relationship between them. The samples generated by this algorithm are closer to the truth, so it can yield better generalization. However, this algorithm is not suitable for the non-negative features. In this paper, we propose an improved MtM transformation algorithm, which can meet non-negative properties.

## 3. LNMF and MtM Transformation

**Figure 1:**
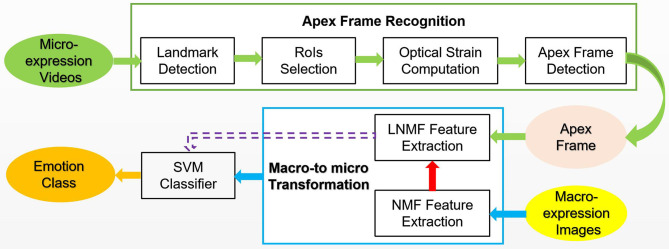
The overall block diagram of the proposed scheme.

The overall scheme is shown in Figure 1. The first row (from left-to-right): the key points of human face are located to cropped eyes and mouth as RoIs, then the optical flow features of RoIs are calculated to detect the apex frame. The second row (from right-to-left): the features of apex frame is extracted from the microexpression videos using LNMF, whereas the NMF is used for extracting the features of macroexpression images. Combined with these two, the proposed MtM transformation is used to increase the samples of microexpression considering the corresponding relationship between macro and micro features. Finally, the classifier based on SVM is trained with all the augmented microexpression samples. In the following, we will discuss the key problems of the proposed scheme about RoIs selection, apex frame detection, LNMF principle, and MtM transformation.

### 3.1. RoIs Selection

**Figure 2:**
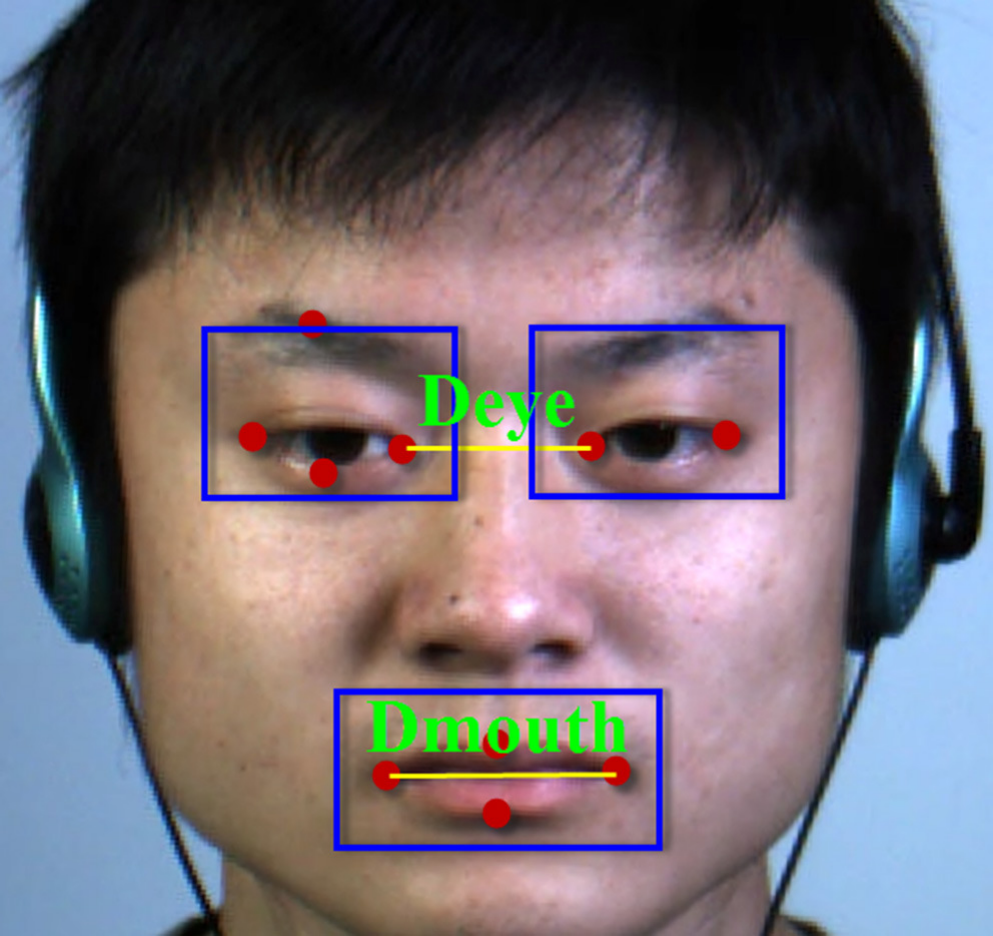
The region of interests (RoIs) of facial expression (Yan et al., 2014).

To determine the RoIs of eyes and mouth regions, we use open source machine learning toolkit DLIB (King, 2009; Ren et al., 2014) to locate the key points of the first frame in the video including microexpression. It should be noted that the position of key points can be assumed unchanged because the face displacement is very small in video. Therefore, we only detect the key points on the first frame. As shown in Figure 2, we use the distance of inner eyes/mouse corners *D*_*eye*_/*D*_*mouth*_, respectively, to determine the RoIs. The distance between the left and right of bounding box of the eyes is *D*_*eye*_/4, the downside is *D*_*eye*_/5 away from the lowest point of the eye, and the topside is located on the highest point of the eyebrow. The left and right of the bounding box of the mouth are *D*_*mouth*_/5 away from the mouth corners, the top is *D*_*mouth*_/4, and the bottom is *D*_*mouth*_/7 from the highest and lowest points of the mouth, respectively.

### 3.2. Apex Frame Detection

The optical flow of a pixel refers to its displacement between two frames, which includes both the horizontal and vertical displacement. The optical strain is calculated as the difference of optical-flow values between pixels, which reflects the deformation degree of a non-rigid body during the motion. The microexpression is the micro movement of facial muscles, and the distortion caused by the movement is reflected by the higher optical strain value of this region.

Let *v*_*x*_ and *v*_*y*_ be the optical flow in horizontal and vertical directions, and the definition of optical strain is expressed as follows:

(1) $$\begin{array}{l} \varepsilon_{m}=[\begin{array}{cc} \varepsilon_{xx}=\frac{\partial v_{x}}{\partial x} & \varepsilon_{yx}=\frac{1}{2}\left(\frac{\partial v_{x}}{\partial y}+\frac{\partial v_{y}}{\partial x}\right)\\ \varepsilon_{xy}=\frac{1}{2}\left(\frac{\partial v_{x}}{\partial x}+\frac{\partial v_{y}}{\partial y}\right) & \varepsilon_{yy}=\frac{\partial v_{y}}{\partial y}) \end{array}] \end{array}$$

(2) $$\begin{array}{l} \varepsilon =\sqrt{\varepsilon_{xx}^{2}+\varepsilon_{yx}^{2}+\varepsilon_{xy}^{2}+\varepsilon_{yy}^{2}} \end{array}$$

where ε_*m*_ contains the normal and tangential strain of the pixel, and ε is the optical strain value of the pixel.

The pseudo codes for the binary search algorithm to detect the apex frame (Liong et al., 2015) are shown in Algorithm 1. First, we calculate the sum of the optical strains of the pixels in each RoI of all frames and take them as the apex frame range in *Iteration*1. Then, we separate the candidate frames into two average parts, and compare the sum of the optical strains values. The larger one will be the candidate range for the next iteration. Afterward, we repeat the calculation until one frame is converged, that is, the detected apex frame.

**Algorithm 1:** Binary Apex Frame Detection.

**input:** {ε_*f*_ ∣ f = 1,…N} : Optical strain of every frame
**output:** n : Apex frame No.
1: **function**
2: *result* ← 0
3: *lo* ← 0
4: *hi* ← *N*
5: **while** *lo* < *hi* **do**
6: *mid* ← (*lo* + *hi*)/2
7: *sum*1 ← *sum*(ε_*lo*_, …, ε_*mid*_)
8: *sum*2 ← *sum*(ε_*mid*_, …, ε_*hi*_)
9: **if** *sum*1 ≤ *sum*2 **then**
10: *lo* ← *mid*
11: **else**
12: *hi* ← *mid*
13: **end if**
14: **end while**
15: **return** *lo*
16: **end function**

### 3.3. Local Non-negative Matrix Factorization

The definition of NMF is expressed as Equation (3):

(3) $$\begin{array}{l} D=WH \end{array}$$

where *D* ∈ *R*^*m*×*n*^ is data matrix; *H* ∈ *R*^*r*×*n*^ is coefficients matrix, in which each column is one sample; and *W* ∈ *R*^*m*×*r*^ is base matrix, in which each column is a base. Define *Y* = *WH*. NMF takes KL divergence as loss function to measure the effect of factorization as follows:

(4) $$\begin{array}{l} D\left(X||Y\right)=\underset{i,j}{\sum }\left(x_{ij}\log\frac{x_{ij}}{y_{ij}}-x_{ij}+y_{ij}\right) \end{array}$$

Here, NMF aims to solve the following optimization problem:

(5) $$\begin{array}{l} \;\underset{W,H}{\min}D\left(X||WH\right)\\ s.t.W,H>0,\underset{i}{\sum }w_{ij}=1\forall j \end{array}$$

In the optimization process, only non-negative constraints are imposed without local constraint to *W*. The learned bases are redundant, and the samples cannot be decomposed into individual components. Nevertheless, the LNMF can solve this problem, where three constraints are added: (i) The base should be indivisible, so the sum of squares of elements in the base should be as small as possible. (ii) The bases should be as orthogonal as possible to reduce redundant information. (iii) It is hoped that the most important information in the original data will be retained in *W*, and the sum of squares of *H* column elements will be as large as possible. Define *U* = *W*^*T*^*W* and *V* = *HH*^*T*^, then the optimization function of LNMF is expressed as follows:

(6) $$\begin{array}{l} \underset{W,H}{\min}D\left(\left(X||WH\right)+\alpha \underset{i,j}{\sum }u_{ij}-\beta \underset{i}{\sum }v_{ii}\right) \end{array}$$

where α and β are constants >0. LNMF iteratively solves Equations (7–9).

(7) $$\begin{array}{l} H=\sqrt{H*\left(W^{T}\frac{D}{WH}\right)} \end{array}$$

(8) $$\begin{array}{l} w_{kl}=\frac{{\left(W*\left(\frac{D}{WH}H^{T}\right)\right)}_{kl}}{\sum_{j}h_{lj}} \end{array}$$

(9) $$\begin{array}{l} w_{kl}=\frac{w_{kl}}{\sum_{k}w_{kl}} \end{array}$$

where “product” means Hadamad product and “division” means matrix division calculation element by element.

**Figure 3:**
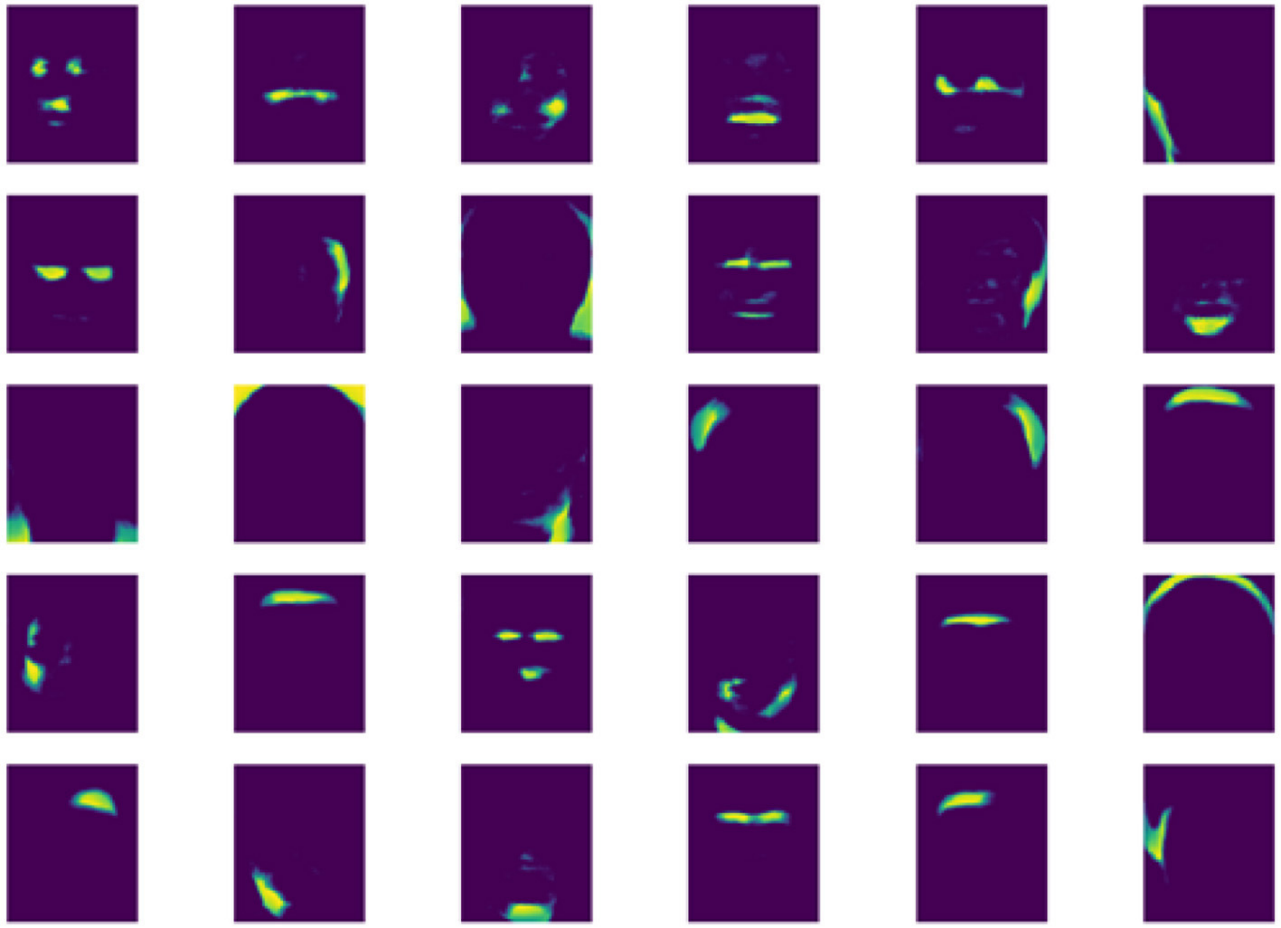
Base matrix of local non-negative matrix factorization (LNMF) for apex frame.

The base matrix *W* obtained from LNMF is shown in Figure 3. It can be seen that each base is sparse, only representing the small area of the face. The microexpression is composed of these areas, which further verifies that LNMF is suitable for microexpression feature extraction. The columns of *H* are the extracted features. We use LNMF for the three RoIs of human face, that is, left/right eyes and mouth, respectively. Finally, we concatenate the three *H* as the features of samples. The advantage is that we can choose different feature dimensions for eyes and mouth to extract more sufficient features for face action. Obviously, more complex movement pattern of eyes corresponds to a higher dimension.

### 3.4. Macro-to-Micro Transformation

The fewer samples in the existing microexpression datasets are usually insufficient to train a classifier with good generalization. Jia et al. (2018) proposed an MtM transformation algorithm, which uses macroexpression data to generate new microexpression samples. The basic principle is described as follows:

$$M=[\begin{array}{ccc} X_{ref}^{1} & \cdots & X_{ref}^{m}\\ Y^{1} & \cdots & Y^{m} \end{array}]$$

where *X*/*Y* represents macro-/microexpression feature sets, respectively; *X* can be decomposed into *X*_*ref*_ and *X*_*probe*_; and $X_{ref}^{i}$ and *Y*^*i*^ represent the same type of microexpression emotions. The SVD of *M* is given as:

(10) $$\begin{array}{l} M=USV^{T} \end{array}$$

where *U* can be expressed as:

(11) $$\begin{array}{l} U=\left[\begin{array}{c} R_{x}\\ R_{y} \end{array}\right] \end{array}$$

where *R*_*x*_/*R*_*y*_ corresponds to macro-/microexpressions in *M*, respectively. It is used to calculate the weighted sum of column vectors of *R*_*x*_ to get $X_{ref}^{i}$ (the *i*th sample in *X*_*ref*_), and the sum of column vectors of *R*_*y*_ with same weights to get *Y*_*i*_ (the *i*th sample in *Y*_*i*_), respectively. That is, if we use *R*_*x*_ to get a macroexpression feature, we can also use *R*_*y*_ to get a microexpression feature. So we have:

(12) $$\begin{array}{l} X_{probe}=R_{x}H \end{array}$$

(13) $$\begin{array}{l} Y_{new}=R_{y}H \end{array}$$

where *H* is weight matrix, *Y*_*new*_ is new microexpression feature samples, and the microexpression emotion is same as each column of *X*_*probe*_.

Because the new feature samples generated by this algorithm do not have non-negative properties, they cannot be used for feature extraction based on LNMF. The reasons include that *U* is an orthogonal matrix, and in order to meet the requirements of orthogonality, it is impossible that every element is a non-negative number, namely *H* must have negative elements. In addition, the method deriving *H* involves the calculation of inverse matrix. When the determinant of matrix is close to 0, the result is not accurate. To acquire the non-negative features,*R*_*x*_, *R*_*y*_, and *H* must be non-negative. Let *R*_*x*_ be the NMF features of macroexpression, and *R*_*y*_ be the LNMF features of microexpression. *H* is derived by NMF method, so we can get an improved MtM algorithm. The pseudo codes of the proposed algorithm are shown in Algorithm 2.

**Algorithm 2:** Macro-to-Micro Transformation.

**input: X**: features of macroexpression; **Y**: features of microexpression
**output**: RES: new features of microexpression
1: **function**
2: *RES* ← {}
3: **for all** *emo* **do**
4: **X**_*emo*_ ← extract features of emo from **X**;
5: **Y**_*emo*_ ← extract features of emo from **Y**;
6: split **X**_*emo*_ into **X**_*emo,ref*_ and **X**_*emo,probe*_
7: calculate **H**_*emo*_ using Equation (16), iteratively
8: **Y**_*new*_ ← **Y**_*emo*_**H**_*emo*_
9: *RES* ← *RES*⋃**Y**_*new*_
10: **end for**;
11: **return** *RES*
12: **end function**

Let *X*_*emo*_ represents the macroexpression NMF feature set of *emo* emotion, which is deposed into *X*_*emo,ref*_ and *X*_*emo,probe*_. Let *Y*_*emo*_ represents the LNMF feature sample set of microexpression, and the columns number is same as *X*_*emo,ref*_. Then we use *X*_*emo,ref*_ to derive the linear representation of *X*_*emo,probe*_:

(14) $$\begin{array}{l} X_{emo,probe}=X_{emo,ref}H_{emo} \end{array}$$

(15) $$\begin{array}{l} Y_{emo,new}=Y_{emo}H_{emo} \end{array}$$

Equation (16) solves *H*_*emo*_ from Equation (14) with NMF formula of fixed *W*:

(16) $$\begin{array}{l} H_{emo}=H_{emo}*\frac{{\left[X_{emo,ref}^{macro}\right]}^{T}X_{emo,probe}^{macro}}{X_{emo,ref}^{macro}X_{emo,probe}^{macro}H_{emo}} \end{array}$$

## 4. Experimental Results and Analysis

In this section, we will evaluate the proposed scheme, including experiment overview, SVM classifier selection, dimension optimization on LNMF, experiments on CK+/CASME2/SAMM datasets, and result analysis.

### 4.1. Experiment Overview

In general, researchers often take the predicted emotion classes of microexpressions as recognition objects (Jia et al., 2018), such as disgust, happy, sadness, surprise, and so on. We also adopt this approach. However, it is worth noting that Davison et al. (2018a) and Guo et al. (2019) classify microexpressions using facial action units, instead of predicted emotions to remove the potential bias of human reporting.

Next, we will validate the proposed scheme based on CK+ macroexpression dataset (Kanade et al., 2000; Lucey et al., 2010), CASME2 (Yan et al., 2014), and SAMM (Davison et al., 2018a,b) microexpression datasets. In the experiments, the SVM classifier is used for classifying microexpressions. The optimized dimension on LNMF can contribute to the recognition accuracy of microexpression. The pretest on CK+ is to verify that macroexpression features extracted by NMF are suitable for MtM transformation. The tests on CK+/CASME2 and CK+/SAMM validate that the proposed MtM transformation can improve the recognition accuracy and generalization. The algorithm evaluation compares the performance of the proposed MtM algorithm with original MtM/MDMO/TICS on CASME2, and SA-AT/ATNet/OFF-ApexNet on SAMM.

### 4.2. SVM Classifier

We adopt the SVM classifier from the Sklearn toolbox based on LIBSVM (Chang and Lin, 2011) to test the macro- and microexpression recognition accuracy. In training phase, the leave-one-sample-out (LOSO) cross-validation is adopted. For each fold, all samples from one subject are used as a testing set and the rest is for training. The final recognition accuracy is the average of five test runs. Microexpression recognition is a multiclassification problem. We use one-vs-one trajectory based on SVM binary classifier to train one SVM between any two classes. If the sample includes *n* classes, then we have *n* * (*n* − 1)/2 SVM. The classification result is determined by all the SVM voting together. To classify the linearly inseparable microexpression, we adopt poly after evaluating sigmoid, radial basis function (RBF), and poly kernel functions. Its definition is expressed as Equation (17).

(17) $$\begin{array}{l} K\left(x_{i},x_{j}\right)={\left(\gamma x_{i}^{T}x_{j}+\alpha \right)}^{d} \end{array}$$

where *x*_*i*_, *x*_*j*_ are the feature vectors, and γ, α, *d* are preset hyperparameters.

**Remark 1**. We acquire the optimized parameters about SVM empirically, which are γ = 4, α = 0, *d* = 4 for CASME2, and γ = 4, α = 0, *d* = 1 for SAMM. We just compare the final recognition accuracy with the related references, where the detailed parameter values about SVM cannot be found.

### 4.3. Dimension on LNMF

If the dimension is too small, microexpression features cannot be decomposed into various detailed components based on LNMF. While the dimension is too large, the features will be too scattered. We determine the optimized value through prior testing and comparing with different dimension setting of eyes and mouth. As shown in Table 1, the optimized dimension is 40/40/80 (left/right eyes and mouth) based on CASME2 with a recognition accuracy of 72.6%. Adopt this same approach, we get the optimized dimension 120/120/110 based on SAMM with a recognition accuracy of 74.68%.

**Table 1:** Dimensions and recognition accuracy on local non-negative matrix factorization (LNMF).

		**Dimension on eyes**			
		**40**	**50**	**60**	**70**
**Dimension on mouth**	60	0.694	0.701	0.692	0.720
	70	0.708	0.698	0.702	0.702
	80	**0.726**	0.706	0.707	0.697
	90	0.700	0.690	0.700	0.680
	100	0.719	0.696	0.707	0.706

*Optimal value is bold*.

### 4.4. CK+-Based Pretest

**Figure 4:**
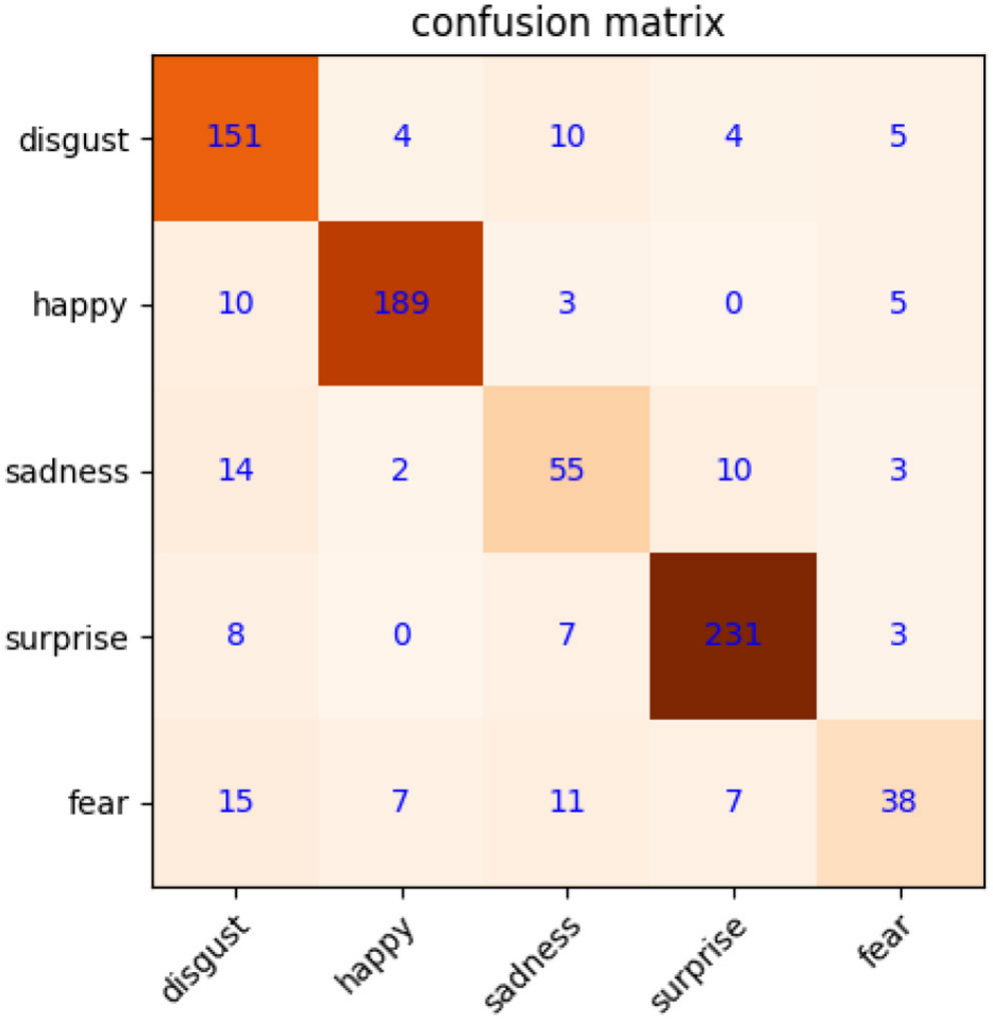
Confusion matrix of macroexpression recognition on CK+.

The precondition for MtM transformation is that macroexpression features have better distinguishability. To validate this, we first calculate the weight coefficients of macroexpression, and then use them to extract the features of macroexpressions based on NMF. The image resolution of CK+ is 48 × 48. We use NMF of 200 dimensions to acquire the features directly. The confusion matrix about macroexpression recognition is shown in Figure 4, with a high accuracy of 83.8%. It shows that macroexpression features extracted by NMF are suitable for MtM transformation to augment microexpression samples.

### 4.5. CK+/CASME2-Based Test

**Figure 5:**
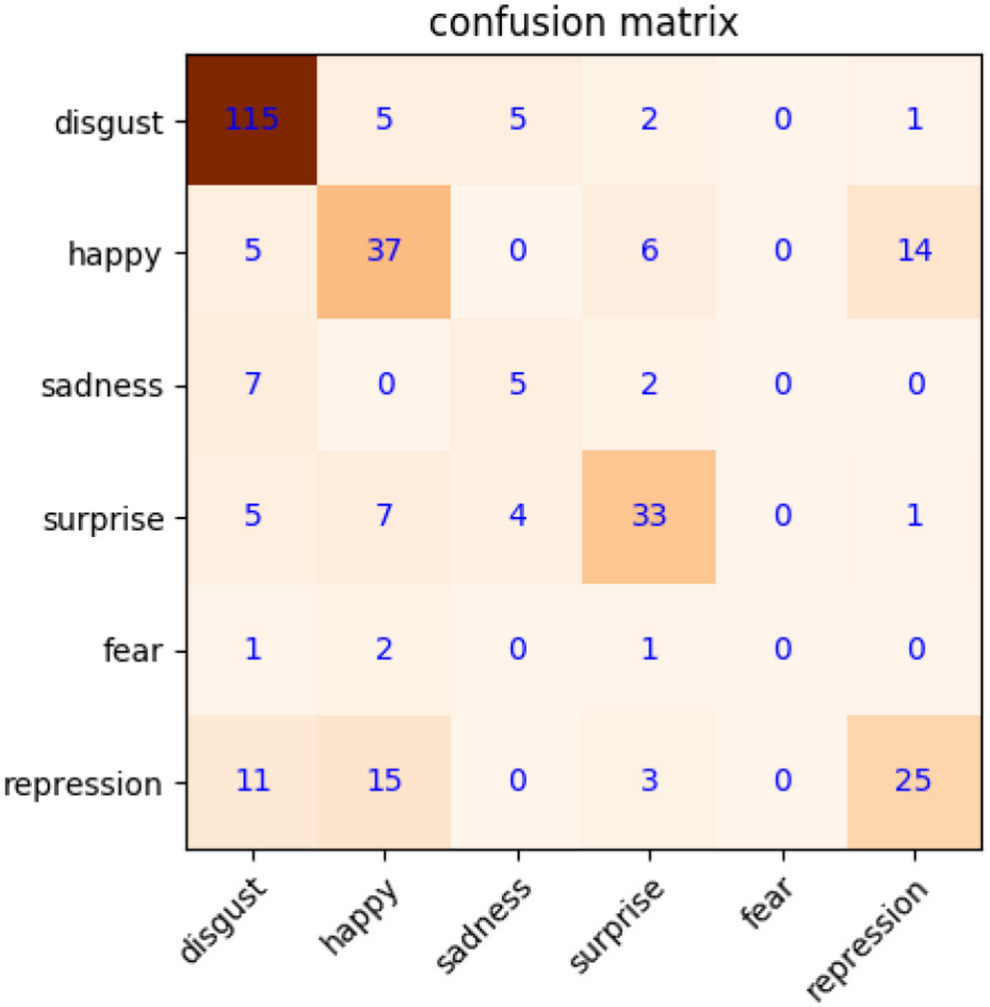
Confusion matrix of microexpression recognition on CASME2 (without new samples).

First, the basic test only focuses on apex frame recognition, LNMF feature extraction, and SVM classifier on CASME2. The RoIs of microexpression are determined according to the distance between inner eyes and mouth corners. It is necessary to normalize the size of eyes to 80 × 90 and mouth to 70 × 150. The 40/40/80 dimension on LNMF is applied to two eyes and mouth regions of samples in CASME2. Three types of features are concatenated in series as the features of CASME2 samples, so the final dimension is 160. Then, the classifier based on SVM is used to test the recognition accuracy by LOSO cross-validation. As shown in Figure 5, the confusion matrix of microexpression with a recognition accuracy of 68.9% (without new samples from our MtM transformation). It will be our baseline compared with the next optimized test.

**Figure 6:**
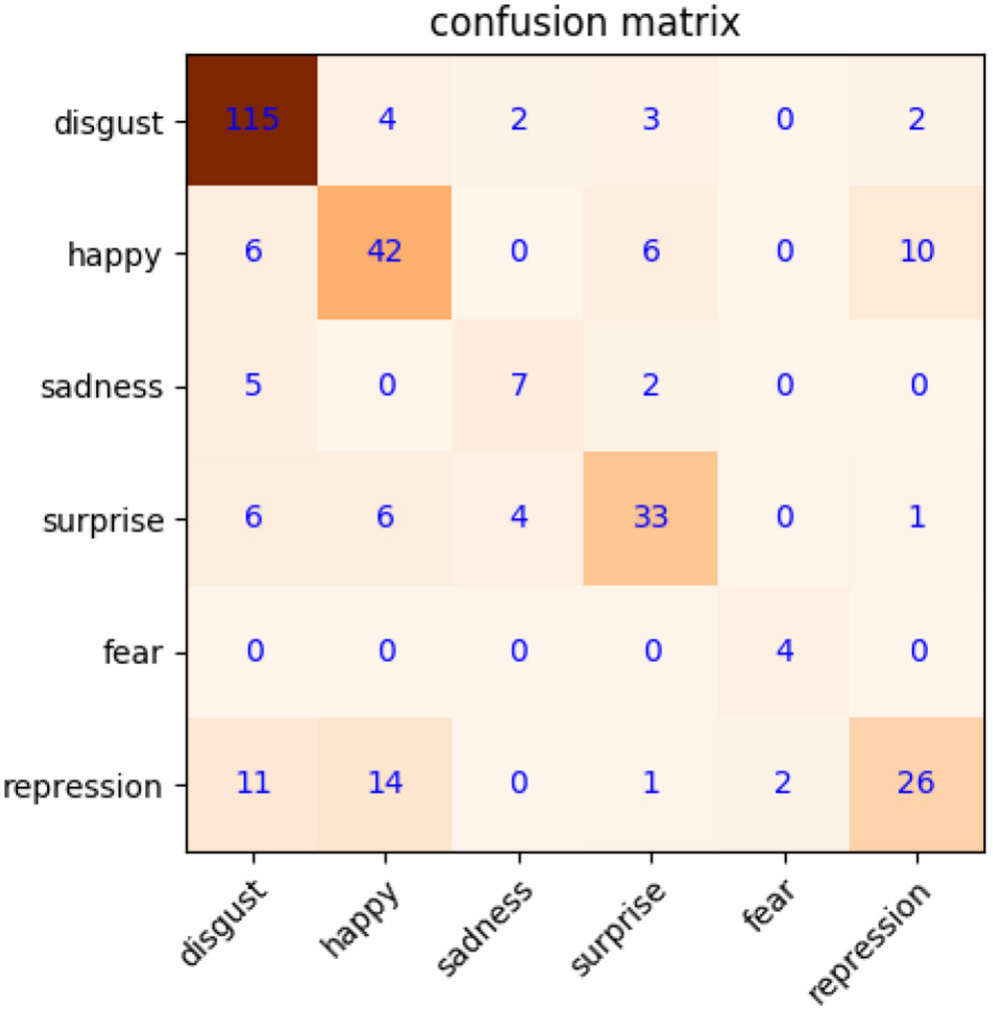
Confusion matrix of microexpression recognition on CK+/CASME2 (with new samples).

Second, the optimized test is carried out with the proposed MtM transformation base on the aforementioned basic test. Considering that CK+ contains anger, contempt, disgust, fear, happy, sadness, and surprise expressions, CASME2 includes disgust, happy, sadness, surprise, fear, repression, and so on. To compare with (Jia et al., 2018) under equivalent conditions, we adopt the same emotions, that is, disgust, happy, sadness, surprise, fear, and repression. In CK+, there are 792 samples labeled as disgust, happy, sadness, surprise, and fear expressions. Moreover, half of the expression samples are separated as *X*_*emo,ref*_ and *X*_*emo,probe*_ for subsequent MtM transformation. There are only 156 samples in CASME2, so we double them to 312 through mirroring. For one-to-one correspondence between microexpression in CASME2 and macroexpression in CK+, we use the samples in CASME2 repeatedly to match macroexpression samples in CK+. By this way, we can acquire 312 original samples and 396 new samples (total of 708) for microexpression recognition. After MtM transformation, we get more microexpress samples, including original, mirrored, and new from MtM transformation. It can contribute to train a better SVM classifier. As shown in Figure 6, the recognition accuracy improves about 3.8–72.7%, compared with Figure 5. It can be seen that the recognition accuracy of happy, sadness, and fear has increased significantly, while surprise and repression unimproved evidently. The reasons actually lie in some similarities, such as eyebrow raising movements (Guo et al., 2020) for surprise, disgust, happy, and sadness expression, and similar cheek movements for repression, disgust, and happy. These similarities cannot be distinguished only through the LNMF features of apex frame, which is a limitation to our algorithm at present, but it can be one of our future working.

However, we only select the original 312 samples in CASME2 for testing, instead of the newly augmented samples (only for training), to avoid distorting the recognition accuracy. Although larger number of new samples can increase the final recognition accuracy, it is not consistent with the fact. We double the samples through mirroring only in training set. When using LOSO cross-validation, it is necessary to exclude the mirrored samples for testing to prevent the false high accuracy caused by two similar samples.

### 4.6. CK+/SAMM-Based Test

There are totally 159 samples in SAMM dataset (Davison et al., 2018b), which includes seven types of emotions, such as anger (57), sadness (6), fear (8), others (26), surprise (15), disgust (9), contempt (12), and happiness (26). To compare performance under equal conditions, we divide the emotions into positive (happiness), negative (anger, sadness, fear, disgust, and contempt) and surprise as the same as Liong et al. (2019), Peng et al. (2019), and Zhou et al. (2019).

**Figure 7:**
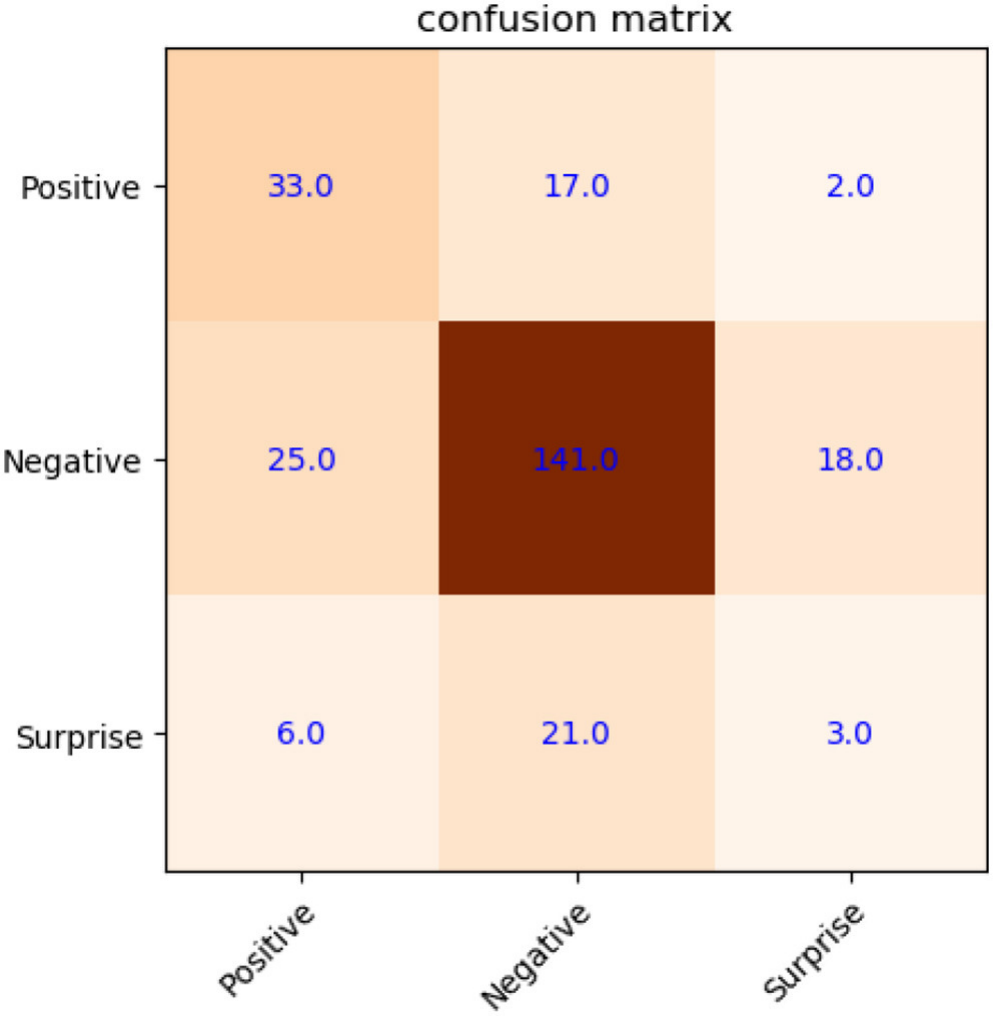
Confusion matrix of microexpression recognition on SAMM (without new samples).

Figure 7 shows the confusion matrix of microexpression with a recognition accuracy of 66.54% (without new samples). Here, only the features on the original and mirrored samples are extracted from SAMM based on LNMF directly, then SVM classifier with LOSO cross-validation is used to classify the microexpressions.

**Figure 8:**
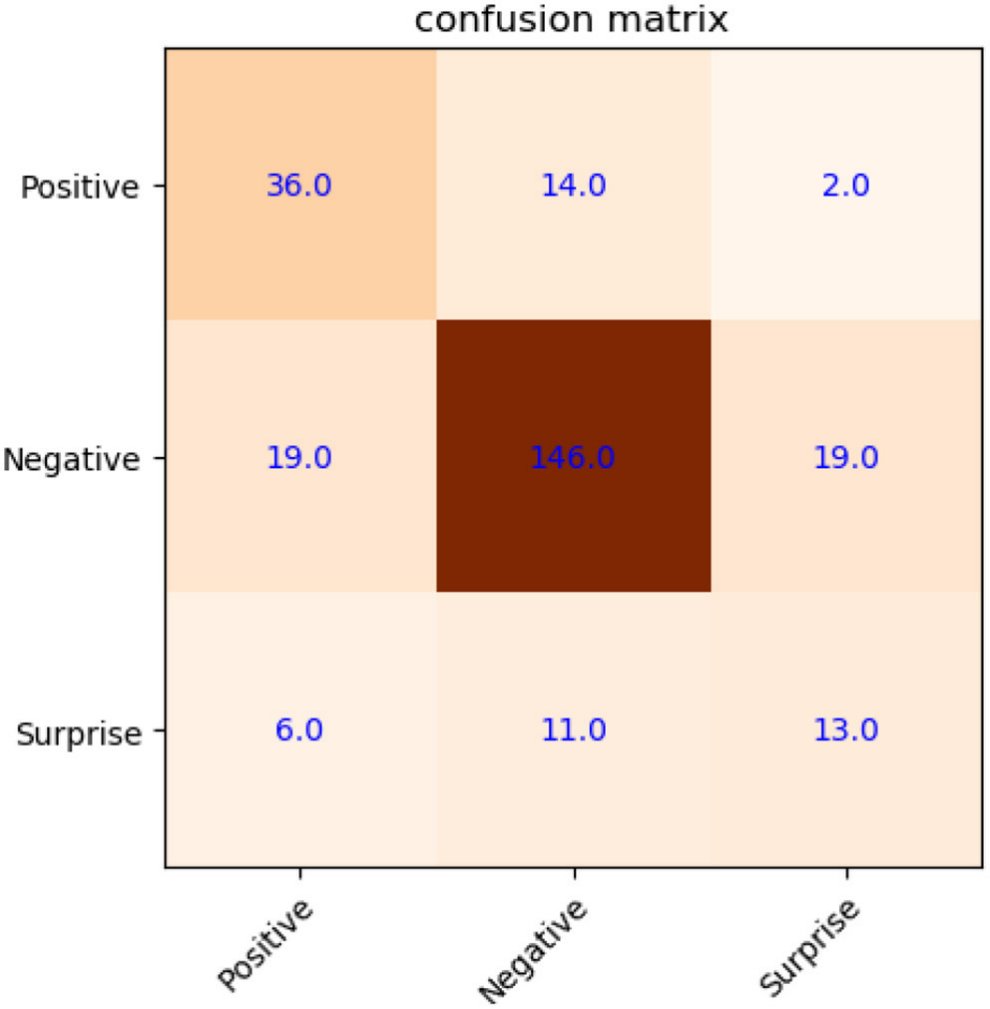
Confusion matrix of micro-expression recognition on CK+/SAMM (with new samples).

Compared with Figure 7, it shows a recognition accuracy of 73.3%, and increased by 6.76%, as shown in Figure 8. Especially in the surprise emotion, its recognition accuracy improved quite a lot with dramatically augmented samples. Here, similar with the experiment on CASME2, three types of samples are used to train the SVM with better generalization in this experiment, including original, mirrored, and new from MtM transformation.

### 4.7. Algorithm Evaluation

We evaluate the proposed MtM algorithm by comparing it with the original MtM (Jia et al., 2018), MDMO (Liu et al., 2016), and TICS (Wang et al., 2014a) based on CASME2, respectively. As shown in Table 2, the proposed MtM algorithm has better performance with a recognition accuracy of 72.6%. Therefore, LNMF can extract more accurate features, and MtM transformation can expand the training samples significantly to prompt the SVM classifier to have better generalization.

**Table 2:** Recognition accuracy of different algorithms on CK+/CASME2.

**Our MtM**	**Original MtM**	**MDMO**	**TICS**
**0.726**	0.655	0.572	0.618

*Optimal value is bold*.

As for SAMM, we evaluate the proposed MtM algorithm by comparing it with SA-AT (Zhou et al., 2019), ATNet (Peng et al., 2019), and OFF-ApexNet (Liong et al., 2019), respectively. As shown in Table 3, the proposed MtM algorithm also has better performance with a recognition accuracy of 73.3%.

**Table 3:** Recognition accuracy of different algorithms on CK+/SAMM.

**Our MtM**	**SA-AT**	**ATNet**	**OFF-ApexNet**
**0.733**	0.549	0.701	0.682

*Optimal value is bold*.

## 5. Conclusion

A new microexpression recognition scheme is proposed, which includes feature extracting and sample expanding. We first determine RoIs with optimized dimensions by facial feature points, then the apex frame is obtained from microexpression video by optical flow method. Afterward, LNMF is developed for each RoI, the results of which are concatenated in series as features of microexpression. Furthermore, the MtM transformation based on LNMF is used, which can increase microexpression samples significantly. A classifier based on SVM is trained with microexpression features and yields better generalization. Finally, the proposed MtM algorithm shows better performance in comparison with other algorithms.

However, the proposed algorithm cannot distinguish some expressions with similar motion features at present. There are obvious recognition confusion on similar eyebrow rising motion, such as surprise, disgust, and happy expression. Therefore, our future work will focus on better feature extraction algorithm to address this issue. Moreover, we will also consider deep forest (Ma et al., 2020; Zhang et al., 2020) as classifier and deep neural networks (Wang et al., 2018) for microexpression recognition in the future.

## Data Availability Statement

The datasets analyzed for this study can be found at CK+: http://www.jeffcohn.net/Resources/, CASME2: http://fu.psych.ac.cn/CASME/casme2-en.php, and SAMM: http://www2.docm.mmu.ac.uk/STAFF/m.yap/dataset.php.

## Ethics Statement

Written informed consent was obtained from the individual(s) for the publication of any potentially identifiable images or data included in this article.

## Author Contributions

JGa, HC, and XZ: conceptualization, methodology, and validation. JGa and HC: software. JGa, HC, XZ, and WL: writing and original draft preparation. JGa, HC, XZ, JGu, and WL: writing–review and editing. All authors have read and agreed to the published version of the manuscript.

## Conflict of Interest

The authors declare that the research was conducted in the absence of any commercial or financial relationships that could be construed as a potential conflict of interest.
